# Comparing The Effectiveness Of Mentalization-Based Therapy And Dialectical Behavior Therapy In An Adult Population With Cluster B Personality Disorders To Reduce Hospital Service Use

**DOI:** 10.1192/j.eurpsy.2024.158

**Published:** 2024-08-27

**Authors:** P. Yin, F.-S. Lahaie, A. Allery, F. Pérusse, L. Cailhol, S. Poirier

**Affiliations:** ^1^Faculty of Medicine, University of Montreal; ^2^Service des Troubles relationnels et de la personnalité, Institut Universitaire en Santé Mentale de Montréal; ^3^Department of Psychiatry and Addictology, University of Montreal, Montreal, Canada

## Abstract

**Introduction:**

Mentalization-based therapy (MBT) and dialectical behavior therapy (DBT) are two treatments known to be effective for borderline personality disorder (BPD). However, head-to-head comparisons between those two treatments are scarce and their effectiveness in naturalistic clinical services, where BPD is often comorbid with other cluster B personality disorders (PD), needs to be further explored.

**Objectives:**

The study’s goal was to answer the following question: Is there a difference in emergency department visits, hospitalizations and dropout rates after one year of treatment in MBT compared to DBT for a clinical adult population with cluster B PD?

**Methods:**

We compared the effectiveness of MBT and DBT in 288 patients between 2015 and 2019 with at least one cluster B PD by measuring their emergency services use and hospitalizations one year before and one year after beginning therapy. Drop-out rates for those two treatment modalities are also compared. Image 1 illustrates the patient distribution for the study.

**Results:**

In terms of reducing emergency room use, patients in each treatment group experienced a significant decrease with medium effect sizes (*p < .001 for both, d=.768 for MBT and d=.640 for DBT*).

In terms of reducing hospitalizations, the MBT group had a significant decrease (*p < .05*) with a medium effect size (*d=.568*) whereas the DBT group had a non-significant decrease (*p = .595*) with a negligible effect size (*d=.140*).

When we compare both therapies, no significant differences were found between them in terms of reductions in emergency room use (*p = .358*) and hospitalizations (*p =.195*), as well as dropout rates (*p = .743*). Image 2 further illustrates the dropout trends in the first year of treatment for both groups in intervals of 3 months.

Hospitalizations were rare in our population, which may hinder the validity of results containing this variable. In absolute numbers, total emergency room visits decreased from 119 to 37, whereas hospitalizations were reduced from 24 to 12. Drop-out rates before entering treatment were high (20.6%), as it was during treatment for both therapies (around 30% in the first year of treatment).

**Image:**

**Figure:**
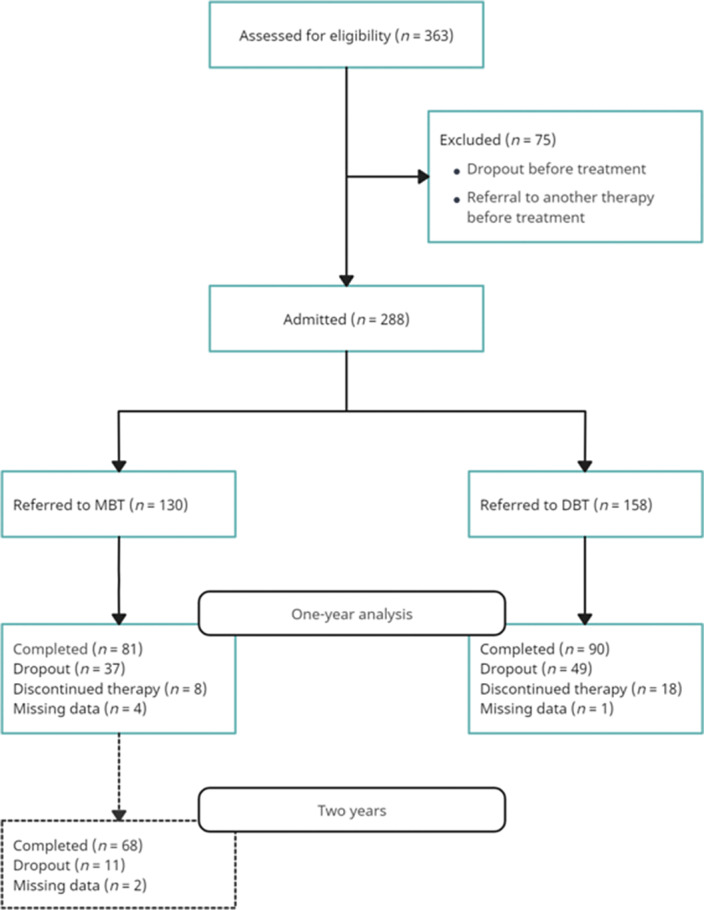

**Image 2:**

**Figure:**
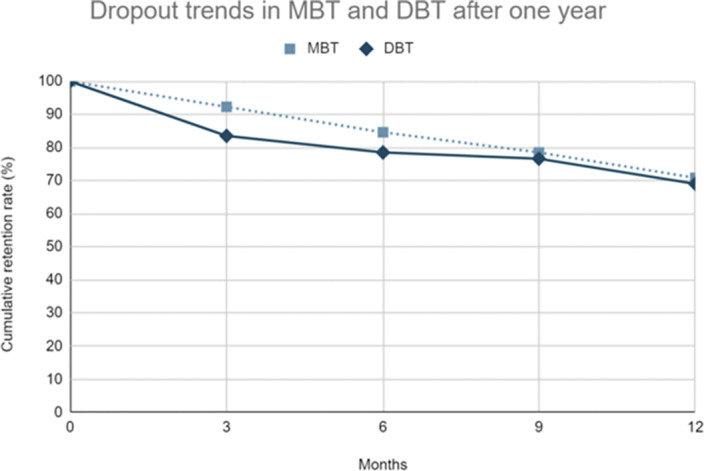

**Conclusions:**

This study emphasizes that both DBT and MBT are linked to a reduction in service use over time. Dropout rates in both treatments are also similar to other studies. Therefore, future research should investigate the factors that can help clinicians guide individuals with PDs towards the type of therapy that is most suitable for them.

**Disclosure of Interest:**

None Declared

